# Treatment of Enlarged Tonsils

**Published:** 1871-06

**Authors:** 


### Treatment of Enlarged Tonsils:

Dr. Rumbold. St. Louis, Mo. (*Med. Archives*), has treated successfully a number of cases of enlarged tonsils by injecting the glands by means of a hypodeamic syringe, with a solution of iodine—iodine gr. ij., potass. iod. ℈ ij., aquæ ℥ j. Generally a slight inflammation followed the injection, but soon subsided. From 12 to 17 injections—ordinarily two a week—were sufficient to reduce the gland to its normal condition. The advantage claimed for this mode of treatment was, saving the substance and function of the gland.

